# Editorial: Diagnosis, epidemiology and treatment of salivary gland carcinomas

**DOI:** 10.3389/fonc.2024.1379584

**Published:** 2024-02-20

**Authors:** Imperia Nuzzolese, Pierluigi Bonomo, Ester Orlandi, Andreas Mock, Stefano Cavalieri

**Affiliations:** ^1^ Head and Neck Medical Oncology Department, Fondazione IRCCS Istituto Nazionale dei Tumori di Milano, Milan, Italy; ^2^ Radiation Oncology, Azienda Ospedaliero-Universitaria Careggi, Florence, Italy; ^3^ Clinical Department, National Center for Oncological Hadrontherapy (Fondazione CNAO), Pavia, Italy; ^4^ Department of Clinical, Surgical, Diagnostic, and Pediatric Sciences, University of Pavia, Pavia, Italy; ^5^ Division of Translational Medical Oncology, National Center for Tumor Diseases (NCT) Heidelberg and German Cancer Research Center (DKFZ), Heidelberg, Germany; ^6^ Institute of Pathology, Ludwig-Maximilians-Universität (LMU) München, Munich, Germany; ^7^ Department of Oncology and Hemato-oncology, University of Milan, Milan, Italy

**Keywords:** salivary gland carcinoma, multidisciplinarity, head and neck, rare cancers, adenoid cystic carcinoma

Salivary gland cancers (SGC) are heterogeneous entities representing less than 5% of head and neck (HN) tumors. The majority of benign SGC are amenable to curative surgery, while the over 20 malignant epithelial SGC subtypes (1) typically require multimodal treatment approaches, combining surgery with radiotherapy to treat local and loco-regional advanced disease. Recent advances in molecular profiling of malignant SGC let to biology-guided treatment paths in the recurrent/metastatic (R/M) setting. While early studies primarily compared adenoid cystic carcinomas (ACCs), one of the most common salivary malignancies, with non-ACC SGCs, a growing list of emerging biomarkers shape a continuously finer grained molecular understanding of the histological subtypes. This Research Topic aimed to promote these developments towards precision oncology trials and new drug developments in SGC. The articles published within the Research topic focused on both ACC and the wide and heterogeneous group of non-ACCs.

On 24 June 2022, in the meeting “Current management and future challenges in salivary gland cancers” held at the National Cancer Center for Oncological hadrontherapy in Italy, several international experts discussed the most significant innovations in molecular profiling, local treatments (i.e., surgery, radiotherapy [RT]), and the development of novel systemic drugs. The expert panel highlighted that it is essential to engage in collaborative research networks to enhance efficiency. Networks play a vital role in facilitating the organization and management of international clinical trials for rare malignancies, such as SGCs. Tailored research plans are needed to foster advancements of care in this setting. The conference proceedings, citing more than 100 articles, contributed to this Research Topic reflecting the rapid evolution of the current SGC scenario, focusing on the exciting progress that has been made in many research domains in the last few years (Locati et al.).

In the pre-operative setting, Wang et al. showed the diagnostic potential of amide proton transfer-weighted (APTw) magnetic resonance imaging adopting endogenous contrast by chemical exchange saturation transfer to indirectly reveal mobile peptides in tissues, which seems to correlate to tumor metabolism. The differences in average and, especially, maximum values of APTw distinguished benign and malignant parotid gland tumors. This may help define the nature of parotid lesions better and rationalize the pre-surgical setting.

Given the relevant discrepancies that emerged in the prognostic evaluation of the current classification systems, researchers focused on better risk stratification, thus considering the nodal status for disease management. The current neck nodal status for major SGC was extrapolated from HN squamous cell carcinomas, with a growing number of studies investigating the need for an adapted lymph node (LN) evaluation method in patients with SGC (2–7).

In a retrospective study, LN metastases significantly affected overall survival and recurrence-free survival in submandibular non-ACC, and the impact was established mainly by the number of positive LNs rather than LN size as defined in the current TNM staging (Wang and Shi).

The heterogeneity of these cancers is further reflected in terms of even unusual clinical behavior, sometimes demonstrated by some more indolent forms, as described by Miserocchi et al., who report a singular case of high-grade transformation of polymorphous adenocarcinoma of the oral floor after 20 years from the primary treatment.

Regarding ACC histology, research is moving towards more detailed knowledge of the disease, and different behaviors were found to be evident according to the primary site of the same histology.

Single-cell RNA sequencing was applied to observe the evolution of individual ACC cells in paracarcinoma and carcinoma tissues. Lin et al. reported their examination of ACC at the transcriptome level, identifying special populations of inter-duct cells and pre-malignant cells that could explain the possible origin of ACC cells and the peculiarly high recurrence rate of this histology.

A single-center retrospective analysis combined with available international databases confirmed recent evidence of a worse prognosis of submandibular ACC compared with parotid ACC, associated with early cervical LN and distant metastases along with rapid progression (Zhou et al.). This behavior may be connected to a high MYB/MYBL1 mutation rate and abnormal upregulation of the phosphatidylinositol-3 kinase pathway, which emerged by analyzing their molecular expression patterns.

Furthermore, in a retrospective analysis from the SEER database based on the number of positive LNs in subjects surgically treated for parotid ACC, Han defined three prognostic categories, thus possibly defining a different treatment plan for high-risk patients.

Regarding the R/M setting, in the last few years, the importance of assessing molecular targets has emerged to drive treatment choices in SGC. A comprehensive meta-analysis including more than 3300 patients showed a diversified prevalence of HER2 positivity (HER2+) ranging from 0% to 43% across sixteen subtypes of SGC (Egeberg et al.). Authors observed a trend towards increasing frequency of HER2+ in cancers derived from salivary gland ducts. As seven different definitions of HER2+ emerged from the evaluated studies, researchers suggested prospective clinical trials to determine the optimal definition of HER2+ based on therapy response in SGC with HER2+.

Moreover, in contrast to previous evidence (8), HER2+ appeared to be a negative prognostic factor in androgen receptor (AR)-positive cancers, at least in the recurrent/metastatic setting. In a retrospective study of 74 subjects with salivary duct carcinoma (SDC) and adenocarcinoma not otherwise specified AR+, Cavalieri et al. showed worse outcomes in HER2+ patients compared to HER2- ones. On the other hand, a non-statistically significant higher risk of developing central nervous system metastases emerged in this cohort, thus deriving the importance of assessing the brain at baseline. A possible crosstalk between the two altered pathways suggests evaluating a treatment combination in the future.

Available data further support comprehensive molecular profiling for a more aggressive form as salivary duct carcinoma (SDC). A collection of patients with AR+, HRAS/PIK3CA co-mutated SDC from a single center experience and a systematic literature search showed multiple targeted treatment strategies and their outcomes. Given the lack of data, Rieke et al. suggested further specific studies to define the best treatment sequences for this disease subtype.

Despite this precision medicine approach, chemotherapy can still have a role in SCG, as demonstrated by Onaga et al. in a subgroup analysis of the retrospective study of 40 patients. The authors demonstrated a favorable efficacy of docetaxel plus cisplatin compared to paclitaxel plus carboplatin, which is confirmed to be mainly not effective in ACC histology.

The eleven papers published in this Research Topic constitute a vital contribution to the field. New interesting results are included, new topics and challenges are approached. In particular, this Research Topic aimed to offer a platform to improve our knowledge of SGC to move their treatment into the future finally but, at the same time, highlighted some controversies present in the current research planning, probably due to the rarity of this disease and the lack of uniformity in the research efforts.

In line with major guidelines (9, 10), the diagnosis must be based on histology and immunohistochemistry findings. Molecular characterization has a supplementary role and can help define poorly differentiated or atypical lesions better and provide information on biological behavior, disease management, and possible targeted treatments.

While some studies focus on this molecular approach, defining possible subtypes of the same histology, others still consider SGC a unique disease. As many of these studies are small and retrospective, we promote an international effort to realize better-designed and prospective trials for the future, which could represent a further step forward to the knowledge of the SGC.

## Author contributions

IN: Conceptualization, Methodology, Writing – original draft, Writing – review & editing. PB: Conceptualization, Methodology, Writing – original draft, Writing – review & editing. EO: Conceptualization, Methodology, Writing – original draft, Writing – review & editing. AM: Conceptualization, Methodology, Writing – original draft, Writing – review & editing. SC: Conceptualization, Methodology, Supervision, Writing – original draft, Writing – review & editing.
